# Supplementary material for: Nickel tolerance is channeled through C-4 methyl sterol oxidase Erg25 in the sterol biosynthesis pathway
Source: PLoS Genet. 2024 Sep 16;20(9):e1011413. doi: 10.1371/journal.pgen.1011413 (PMC11426505; doi:10.1371/journal.pgen.1011413)
Supplement: S1 Fig — (A)H99 cells were serially diluted and spotted onto RPMI and RPMI+Ni at the indicatedconcentrations. The plates were imaged after two days of incubation. (B) The indicated strains with cell density OD600 = 3 were spotted onto Christensen Urea Agar (CUA) plates. The plates were incubated for three days and imaged. Urease activity is indicated by the yellow to pink color change of the media due to alkalization of the media by released ammonia. (C) Two molecules of DMG chelate one molecule of Ni. (D) H99 and ure1Δ with cell density OD600 = 3 were spotted onto Christensen Urea Agar (CUA) plates with increasing concentrations of DMG. The plates were incubated for three days and imaged. (E) The indicated strains were serially diluted and spotted onto RPMI and RPMI+ 250μM Ni plates. The plates were imaged after two days of incubation. (PDF) [file pgen.1011413.s001.pdf]

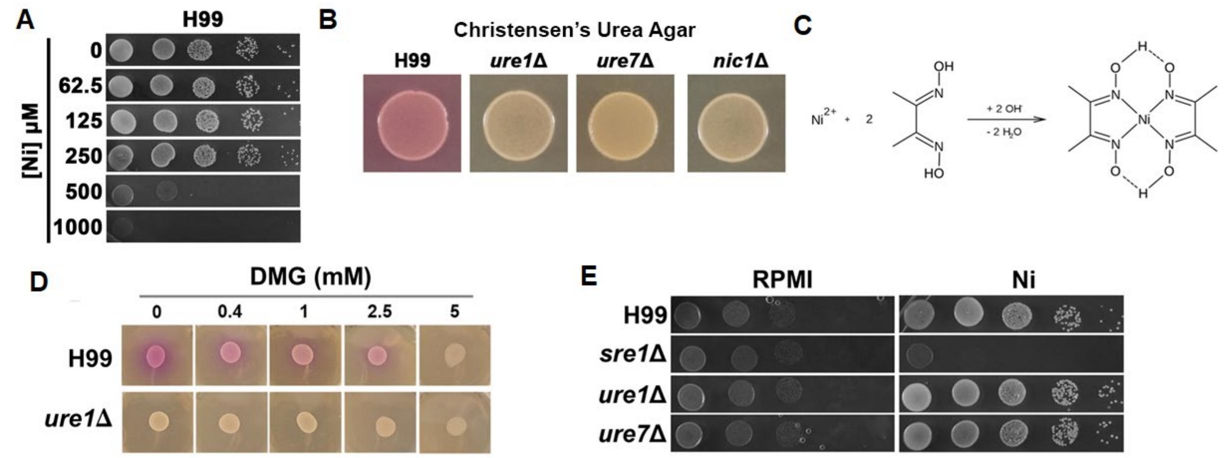


**S1 Fig. Urease is not required for Ni tolerance**

**(A)** H99 cells were serially diluted and spotted onto RPMI and RPMI+Ni at the indicated concentrations. The plates were imaged after two days of incubation. **(B)** The indicated strains with cell density $OD_{600}$=3 were spotted onto Christensen Urea Agar (CUA) plates. The plates were incubated for three days and imaged. Urease activity is indicated by the yellow to pink color change of the media due to alkalization of the media by released ammonia. **(C)** Two molecules of DMG chelate one molecule of Ni. **(D)** H99 and *ure1*Δ with cell density $OD_{600}$=3 were spotted onto Christensen Urea Agar (CUA) plates with increasing concentrations of DMG. The plates were incubated for three days and imaged. **(E)** The indicated strains were serially diluted and spotted onto RPMI and RPMI+ 250µM Ni plates. The plates were imaged after two days of incubation.
