# Supplementary material for: Nickel tolerance is channeled through C-4 methyl sterol oxidase Erg25 in the sterol biosynthesis pathway
Source: PLoS Genet. 2024 Sep 16;20(9):e1011413. doi: 10.1371/journal.pgen.1011413 (PMC11426505; doi:10.1371/journal.pgen.1011413)
Supplement: S2 Fig — (A) The indicated strains were plated onto RPMI media with or without Ni, and incubated at 37°C for 2 days. (B) ICP-MS quantification of Cu (left) and Zn (right) concentrations in cells grown on RPMI+Ni. The same dry weight of cells was used for the analysis. Student’s t-test was used for statistical analysis. **: p ≤0.01, ***: p ≤0.001. (PDF) [file pgen.1011413.s002.pdf]

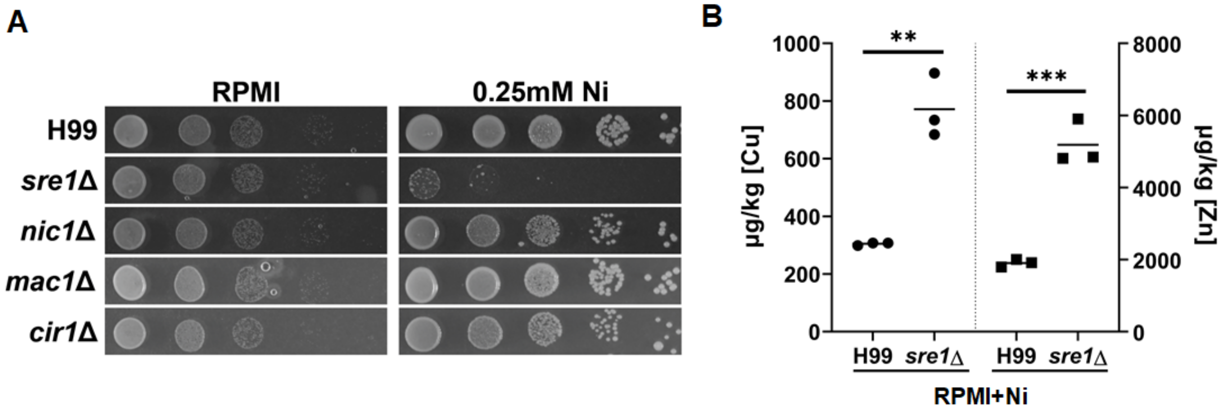


**S2 Fig. Metal accumulation does not dictate metal sensitivity. (A)** The indicated strains were plated onto RPMI media with or without Ni, and incubated at 37°C for 2 days. **(B)** ICP-MS quantification of Cu (left) and Zn (right) concentrations in cells grown on RPMI+Ni. The same dry weight of cells was used for the analysis. Student's *t*-test was used for statistical analysis. **: p ≤0.01, ***: p ≤0.001
