# Supplementary material for: Nickel tolerance is channeled through C-4 methyl sterol oxidase Erg25 in the sterol biosynthesis pathway
Source: PLoS Genet. 2024 Sep 16;20(9):e1011413. doi: 10.1371/journal.pgen.1011413 (PMC11426505; doi:10.1371/journal.pgen.1011413)
Supplement: S3 Fig — (A) Volcano plot of transcript changes of sre1Δ grown on RPMI+Ni versus H99 grown on RPMI+Ni based on RNA-seq data. (B) Volcano plot of transcript changes of sre1Δ grown on RPMI+hypoxia versus H99 grown on RPMI+hypoxia based on RNA-seq data. The red dots in both panels indicate EBP genes present in the data set. (C) Plot of transcript changes of genes shared between H99 Ni vs RPMI and H99 hypoxia vs normoxia data sets. Red highlighted genes are EBP genes. In all panels, dots that fall outside of the shaded grey areas are DEGs. (PDF) [file pgen.1011413.s003.pdf]

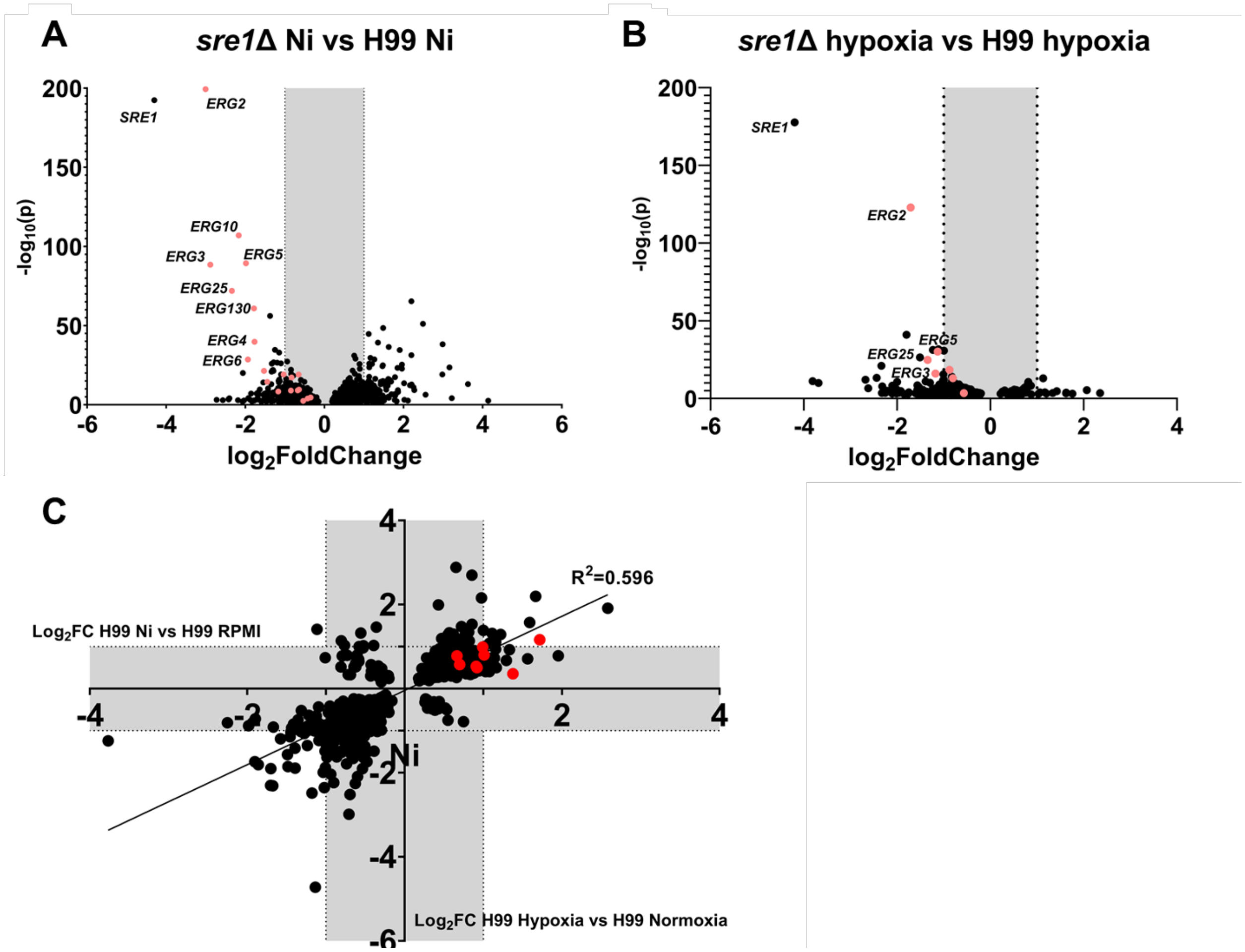


**S3 Fig. Ni and hypoxia have distinct impacts on cryptococcal transcriptome. (A)** Volcano plot of transcript changes of *sre1*Δ grown on RPMI+Ni versus H99 grown on RPMI+Ni based on RNA-seq data. **(B)** Volcano plot of transcript changes of *sre1*Δ grown on RPMI+hypoxia versus H99 grown on RPMI+hypoxia based on RNA-seq data. The red dots in both panels indicate EBP genes present in the data set. **(C)** Plot of transcript changes of genes shared between H99 Ni vs RPMI and H99 hypoxia vs normoxia data sets. Red highlighted genes are EBP genes. In all panels, dots that fall outside of the shaded grey areas are DEGs.
