# Supplementary material for: Nickel tolerance is channeled through C-4 methyl sterol oxidase Erg25 in the sterol biosynthesis pathway
Source: PLoS Genet. 2024 Sep 16;20(9):e1011413. doi: 10.1371/journal.pgen.1011413 (PMC11426505; doi:10.1371/journal.pgen.1011413)
Supplement: S4 Fig — (A) ICP-MS data showing intracellular iron concentrations in H99 and sre1Δ on RPMI (C), RPMI+250μM Ni (N), and RPMI+DMG (D). Student’s t-test was performed to assess statistical significance. ** = ≤0.01, ns = not significant. (B) The indicated strains were serially diluted and spotted onto RPMI and RPMI+250μM Ni media. Plates were incubated at 37°C for two days prior to imaging. (PDF) [file pgen.1011413.s004.pdf]

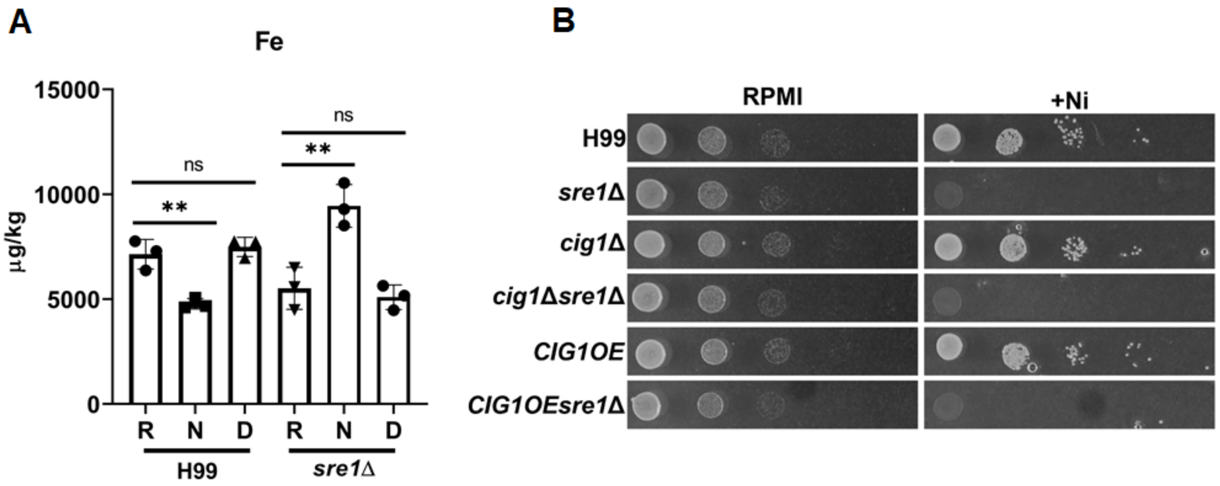

S4 Fig. The *sre1*Δ sensitivity to Ni is not due to iron starvation. (A) ICP-MS data showing intracellular iron concentrations in H99 and *sre1*Δ on RPMI (C), RPMI+250µM Ni (N), and RPMI+DMG (D). Student's *t*-test was performed to assess statistical significance. **= ≤0.01, ns = not significant. **(B)** The indicated strains were serially diluted and spotted onto RPMI and RPMI+250µM Ni media. Plates were incubated at 37°C for two days prior to imaging
