# Supplementary material for: Nickel tolerance is channeled through C-4 methyl sterol oxidase Erg25 in the sterol biosynthesis pathway
Source: PLoS Genet. 2024 Sep 16;20(9):e1011413. doi: 10.1371/journal.pgen.1011413 (PMC11426505; doi:10.1371/journal.pgen.1011413)
Supplement: S5 Fig — (A) Wildtype H99 and sre1Δ cells were serially diluted and spotted onto RPMI, RPMI+250μM Ni, and RPMI+250μM Co. The plates were incubated at 37°C for two days prior to imaging. (B) H99, pas2Δ and sre1Δ cells were serially diluted and spotted onto RPMI, and RPMI with the indicated concentration of Ni. The plates were incubated at 37°C for two days prior to imaging. (PDF) [file pgen.1011413.s005.pdf]

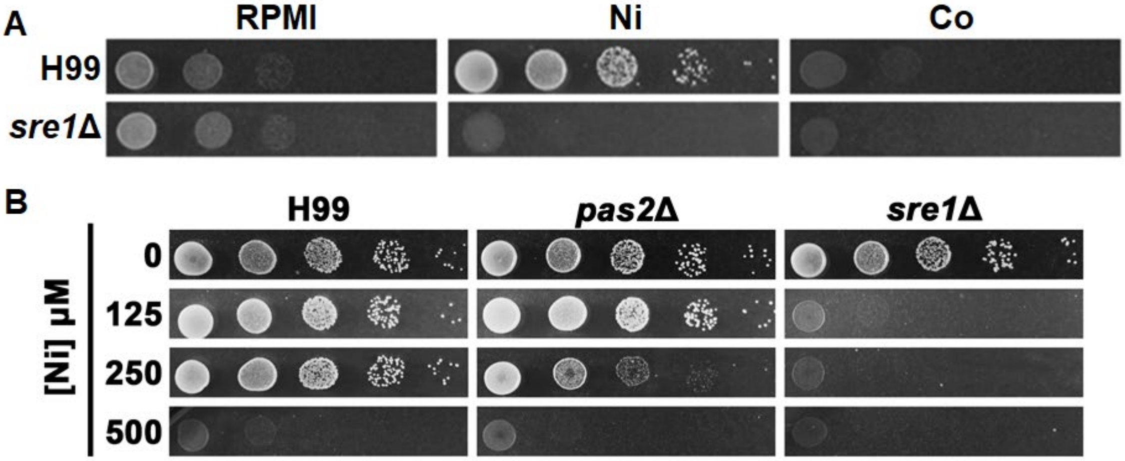

S5 Fig. Ni and Cobalt (Co) elicit overlapping and different effects on growth. (A) Wildtype H99 and *sre1*Δ cells were serially diluted and spotted onto RPMI, RPMI+250μM Ni, and RPMI+250μM Co. The plates were incubated at 37°C for two days prior to imaging. **(B)** H99, *pas2*Δ and *sre1*Δ cells were serially diluted and spotted onto RPMI, and RPMI with the indicated concentration of Ni. The plates were incubated at 37°C for two days prior to imaging.
