# Supplementary material for: Nickel tolerance is channeled through C-4 methyl sterol oxidase Erg25 in the sterol biosynthesis pathway
Source: PLoS Genet. 2024 Sep 16;20(9):e1011413. doi: 10.1371/journal.pgen.1011413 (PMC11426505; doi:10.1371/journal.pgen.1011413)
Supplement: S6 Fig — Cells of the indicated strains were serially diluted and spotted on RPMI and RPMI+250μM Ni. Plates were incubated at 37°C for two days prior to imaging. (PDF) [file pgen.1011413.s006.pdf]

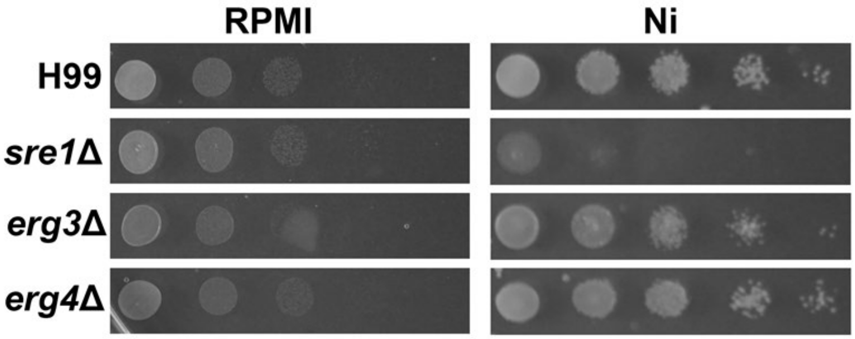

S6 Fig. Neither *ERG3* nor *ERG4* is required for cryptococcal tolerance of Ni. Cells of the indicated strains were serially diluted and spotted on RPMI and RPMI+250µM Ni. Plates were incubated at 37°C for two days prior to imaging.
