# Supplementary material for: Nickel tolerance is channeled through C-4 methyl sterol oxidase Erg25 in the sterol biosynthesis pathway
Source: PLoS Genet. 2024 Sep 16;20(9):e1011413. doi: 10.1371/journal.pgen.1011413 (PMC11426505; doi:10.1371/journal.pgen.1011413)
Supplement: S7 Fig — (A) Cells of H99 with overexpression of the indicated ERG genes were serially diluted and plated onto YNB plates with CoCl2 at the indicated concentration. (B) Cells of the sre1Δ mutant with overexpression of the indicated ERG genes were serially diluted and plated on RPMI plates with the indicated concentrations of CoCl2. (C) Cells of the same strains as in Panel A were spotted onto YPD media and incubated in ambient air (normoxia) or hypoxia (0.1% O2, 5% CO2) conditions. (D) Cells of the same strains as in Panel B were spotted onto RPMI media with the indicated concentrations of Ni. All plates were incubated for two days prior to imaging. (PDF) [file pgen.1011413.s007.pdf]

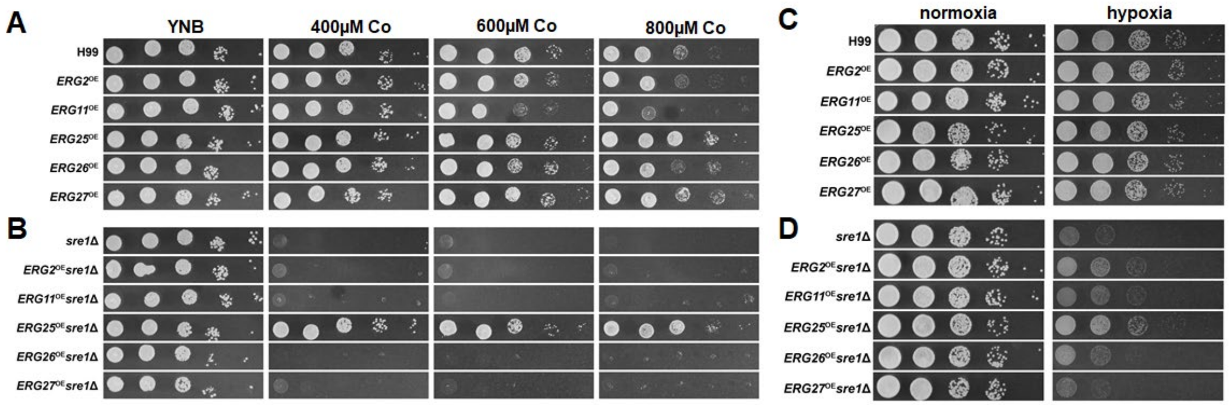

S7 Fig. *ERG25* is required for tolerance of Co and multiple *ERG* overexpression can partially restore the growth defect of *sre1*Δ in hypoxia. (A) Cells of H99 with overexpression of the indicated *ERG* genes were serially diluted and plated onto YNB plates with $CoCl_2$ at the indicated concentration. **(B)** Cells of the *sre1*Δ mutant with overexpression of the indicated *ERG* genes were serially diluted and plated on RPMI plates with the indicated concentrations of $CoCl_2$. **(C)** Cells of the same strains as in Panel A were spotted onto YPD media and incubated in ambient air (normoxia) or hypoxia (0.1% O_2, 5% CO_2) conditions. **(D)** Cells of the same strains as in Panel B were spotted onto RPMI media with the indicated concentrations of Ni. All plates were incubated for two days prior to imaging.
