# Supplementary material for: Nickel tolerance is channeled through C-4 methyl sterol oxidase Erg25 in the sterol biosynthesis pathway
Source: PLoS Genet. 2024 Sep 16;20(9):e1011413. doi: 10.1371/journal.pgen.1011413 (PMC11426505; doi:10.1371/journal.pgen.1011413)
Supplement: S8 Fig — C. neoformans Erg25 protein is 343 amino acids in length. Four histidine enriched putative metal binding motifs are boxed in various colors. The histidine residues predicted to interact with a cation are highlighted in yellow. (PDF) [file pgen.1011413.s008.pdf]

```
>Erg25
MAAAAAFDLLDKYIPGASDSLTIVNATTAQNTLYPGVDFAALNWLERLWA
SYYIWVGNPIIATGLMSFLLHEIVYFGRCIPWLIIDAMPYFQKWKLQPNK
HVSRAQILKCTKVVLLTHFTCEAPLILAFHPICCLFGMKTYEIPFSSIGL
MAAQIAFFFVFEDTFHYWAHRALHFGPLYKHIHKLHHEFSAPIGIAAEYA
HPLEVLILAQGTISGPFLYAVFRDDLHIFTVYVWITLRLWQAVDAHSGYD
FPWSLRHFIPFWAGADHHDFHHATFTSCFSTSFRWWDYYFGTDVKYHAYK
ARVAAASAKERAAVEKNEMERLEKEGILEERAAASHGKRGKNE
```

S8 Fig. Erg25 protein sequence contains histidine residues in predicted metal binding regions. *C. neoformans* Erg25 protein is 343 amino acids in length. Four histidine enriched putative metal binding motifs are boxed in various colors**.** The histidine residues predicted to interact with a cation are highlighted in yellow.
