# Supplementary material for: Nickel tolerance is channeled through C-4 methyl sterol oxidase Erg25 in the sterol biosynthesis pathway
Source: PLoS Genet. 2024 Sep 16;20(9):e1011413. doi: 10.1371/journal.pgen.1011413 (PMC11426505; doi:10.1371/journal.pgen.1011413)
Supplement: S9 Fig — RT-PCR data was generated by harvesting cells from overnight YPD cultures of (A) wild type and URE7OE in the wild type background as well as (B) sre1Δ and sre1ΔURE7OE. Housekeeping gene TEF1 was used as an internal control to ensure the quality of the original RNA sample used for cDNA amplification and for normalization. Student’s t-test was used for statistical analysis. ***: p ≤0.001, ****: p <0.0001. (PDF) [file pgen.1011413.s009.pdf]

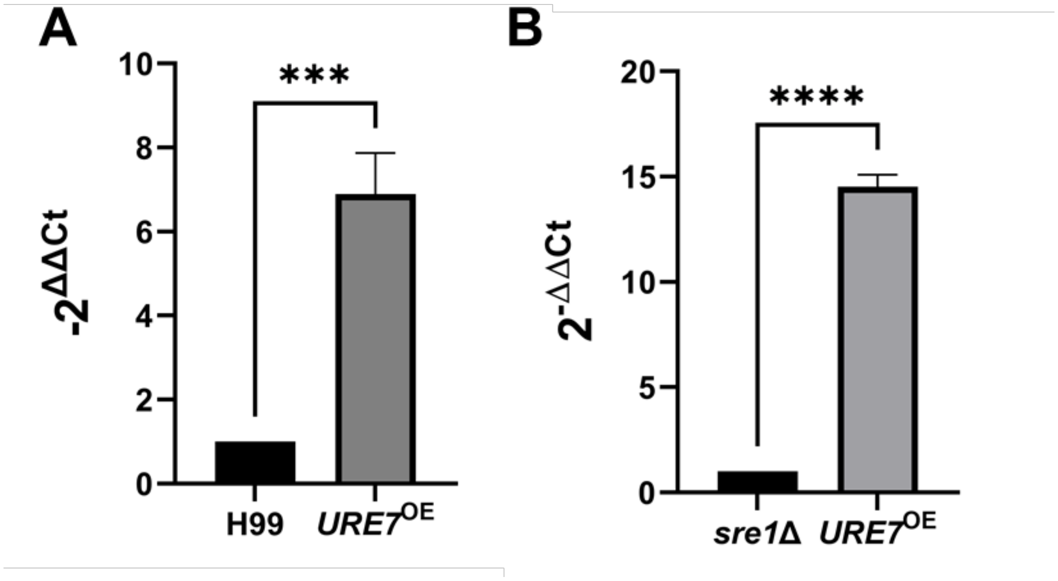

S9 Fig. *URE7* is overexpressed in both wildtype and *sre1*Δ backgrounds. RT-PCR data was generated by harvesting cells from overnight YPD cultures of **(A)** wild type and *URE7*^{OE} in the wild type background as well as **(B)** *sre1*Δ and *sre1*Δ*URE7*^{OE}. Housekeeping gene *TEF1* was used as an internal control to ensure the quality of the original RNA sample used for cDNA amplification and for normalization. Student's *t*-test was used for statistical analysis. ***: $p \leq 0.001$, ****: $p < 0.0001$.
