# Supplementary material for: Nickel tolerance is channeled through C-4 methyl sterol oxidase Erg25 in the sterol biosynthesis pathway
Source: PLoS Genet. 2024 Sep 16;20(9):e1011413. doi: 10.1371/journal.pgen.1011413 (PMC11426505; doi:10.1371/journal.pgen.1011413)
Supplement: Attachment — Submitted filename: Response Letter 8-6-2024-XL clean.pdf [file pgen.1011413.s018.pdf]

Reviewer #1: The authors explore mechanisms of nickel tolerance in the human pathogenic fungus Cryptococcus neoformans. Previous work established that urease enzymes in this microorganism require Ni, but little is known about the cellular response to excess Ni. Using an established collection of strains with mutations in transcription factors, the authors identified the sterol response pathway regulator Sre1 as required for growth in the presence of high concentrations of Ni. This is the major initial finding upon which the rest of the paper builds, and the authors explore various aspects of Sre1 biology and activity to find specific ways Ni is handled in the fungal cell. Fungal Sre1 proteins have been shown to be activated by conditions as distinct as hypoxia and pH, and metal bioavailability may be a common linking theme for Sre1 activation. Interestingly, Ni can also activate the mammalian HIF1 protein, another transcriptional regulator that similarly coordinates hypoxic cellular responses.

The authors' finding is strengthened by their documentation that other proteins involved in Sre1 activation (Scp1, Stp1) are similarly required for Ni tolerance. The authors furthermore explore targets of Sre1, including genes in the ergosterol biosynthetic pathway. The main finding is that Erg25, when overexpressed, increased cryptococcal Ni tolerance, making it somewhat unique among other Erg proteins. Blocking the Er5 pathway itself does not significantly affect Ni tolerance. Also, mutating metal-binding histidine residues results in a poorly functioning protein with regards to Ni tolerance. The reasons for this are not completely clear since the native ERG25 gene is still present. However, the authors offer reasonable explanation in the text.

The authors include a very nice control for their ERG gene overexpression study by documenting increased fluconazole tolerance in these strains.

We thank the reviewer for the kind summary of our work and the highlights of the major findings. We are also grateful for the helpful suggestions to improve the manuscript. Our detailed responses are written below in bold.

Minor points:
1) Line 72. I would reword this sentence to minimize the results placed in the introduction; e.g. "However, the role of urease in C.n. nickel tolerance is unknown" Or similar

The statement in line 72 in the original manuscript has been changed to the suggested statement to reduce the results summarized in the introduction.

2) Line 144. The strain name H99 is used extensively in the manuscript and Figures. It might be more appropriate to simply refer to this strain as "WT", especially in figures.

We thank the reviewer for this suggestion. We replaced "H99" with "wild type" in many occasions (Lines 137, 192, 195, 217, 259, 287, and 324) where appropriate in this revised manuscript.

3) Line 116. The Legend refers to "RPMI+Ni (Ni)" but the figure just has "N" and not "Ni"

This has been corrected in the legend to reflect what is represented in the figure. "Ni" was changed to "N."

4) Line 136. “Fig 1B” should be “Fig 1C”.

This has been corrected.

5) Line 145. I would remove phrases such as “To our surprise” and simply state the results.

We removed such phrases from the text.

6) Line 216. “… much more tolerance …”

Thanks for noticing that. We added “more” to the text.

7) Is it possible to demonstrate whether Sre1 activation (e.g. protein cleavage) is indeed induced by excess Ni?

We thank the reviewer for this suggestion. To verify that Sre1 is indeed activated by Ni, we constructed a Flag-tagged Sre1 strain. We cultured the strain in the absence and the presence of Ni. As a control, we also cultured the strain in the presence of fluconazole as this *ERG11* inhibitor is known to reduce ergosterol level and activate Sre1. We found that Sre1 was indeed cleaved in the presence of Ni and also in response to fluconazole treatment based on our western blot analysis. This newly acquired result is presented as Figure 1C in the revised manuscript and the text in the corresponding result section has been modified accordingly. (lines 136-142)

8) Fig3. Why does Erg11 over-expression result in increased sensitivity to Ni?

We thank the reviewer for noticing this. In *Saccharomyces cerevisiae*, *ERG11* overexpression is known to accumulate 4,4 dimethyl sterols, and sterols that accumulated in this overexpression strain were esterified [1]. Only free sterols can be used by the EBP enzymes. We speculate that when Ni induces a reduction in the amount of ergosterol in the membrane, the sterols that have accumulated in this *ERG11* overexpression strain are incapable of being used. Thus, the strain has a slight growth defect. It is also possible that overexpression of *ERG11* may lead to accumulation of sterol intermediate products when Erg25’s function is impaired by the presence of nickel, which reduces cryptococcal fitness.

9) Line 308. The authors link filipin straining intensity very strongly to ergosterol concentrations. Perhaps they could refer to a 50% decrease in filipin staining, consistent with the lower ergosterol levels measured spectrophotometrically.

We agree with the reviewer about modifying the way the sentence is structured. Now the sentence reads as follows: “Filipin staining of ergosterol present in the outer leaflet of plasma membrane (59, 62, 63) showed that Ni exposure caused a 50% reduction in plasma membrane ergosterol in H99 (Fig 4B-C), consistent with the lower ergosterol levels in the presence of nickel measured spectrophotometrically.” (Lines 288-291)

10) Fig 4. Many of the abbreviations in the graphs are not defined in the legend.

We thank the reviewer for bringing this to our attention. We have modified the legend and defined all the abbreviations. For panel A the legend now reads “RPMI (C), RPMI+250µM Ni (Ni), or RPMI+4µg/mL Flu (F).” For panel C the legend now reads “RPMI (R), RPMI+ 250µM Ni (N).” For panels D and E, we noted the legend that “-” means RPMI media alone and “+” means RPMI+Ni .

11) Line 400. Filipin is a surrogate for PM cholesterol. Therefore, I would change “indicates” to “suggests”.

This change has been made.

12) Line 398. What is the significance of observing desmosomes in these mammalian cells?

In our experience, the appearance of desmosomes indicates healthy cells that are able to grow and develop. Mammalian cells in spent media or media that is not conducive for growth will appear round. Cells lacking desmosomes are also less adhesive to the growth plate. The observation of desmosomes suggests that the cells are healthy.

Reviewer #2: The authors investigate whether nickel overload can influence Cryptococcus neoformans growth and identify a link with sterol regulation, implicated Sre1 and Erg25 as mediating nickel stress resistance. While there are some interesting links between nickel and hypoxia sensitivity identified, and some possible mechanisms proposed through activity of Erg25, these are not fully explored or directly demonstrated. For example, in many places, there is an over-reliance on shared phenotypic outcomes. The work overall is hypothesis generating, rather than a conclusive dissection of function.

We thank the reviewer for taking time to review our manuscript and for the helpful comments and suggestions. We agree with the reviewers that our observations could be explained by two likely hypotheses: Hypothesis one: Erg25 acts as a nickel sink to increase cryptococcal tolerance to nickel. Hypothesis two: specific impairment on Erg25’s function by nickel renders *Cryptococcus* sensitive to nickel. To distinguish these two possibilities, we overexpressed the Ni-chaperone *URE7* as it is known to bind nickel. If Erg25 simply acts as a nickel sink, we would expect that overexpression of *URE7*, like the overexpression of *ERG25* would be capable of restoring growth of *sre1*Δ on Ni. We found that the *URE7* overexpression was incapable of rescuing *sre1*Δ on Ni. We confirmed that this construct was capable of complementing the *ure7*Δ mutant on Christensen Urea Agar and thus functional. Therefore, Ni-binding ability in itself is not sufficient to cause the observed phenotypic effects. The results of this experiment are summarized in the manuscript at lines 352-364. The new data is now presented as Figure 6 in this revised manuscript. We also discussed these hypotheses more extensively in this revision (lines 419-425).

The newly acquired data and also data presented in the original manuscript (e.g. nickel profoundly and more specifically affects EBP pathway; no other ERG genes tested,

including Erg3 with similar metal binding pocket, can confer nickel tolerance) collectively strongly support our hypothesis that nickel specifically targets Erg25 enzyme, which is not simply acting as a nickel sink. Although there are more unanswered questions raised by our observations presented in this study, they provide a good foundation to future investigations as little is known about mechanism regulating nickel homeostasis and nickel toxicity, which is in contrast to extensive investigations and vast knowledge known about copper or iron regulation.

While the explanation that Erg25 over-expression can mediate nickel resistance is an interesting insight, and the attempts to predict how changes in Erg25 metal binding would impact function have potential, this reviewer remains unconvinced that nickel directly impacts Erg25 activity in WT or sre1Δ cells. The alternate interpretation that the observed rescue in Erg25OE cells is mediated by a parallel suppressor activity, for example acting as a nickel sink independent of its native function, does not appear to have been considered. Given that Erg25 acts in a complex with two other EBPs, it remains unclear how Erg25 alone could be influencing nickel and ergosterol-related outcomes.'

We thank the reviewer for this suggestion. Please see our earlier response on the two hypotheses and the results that led to our conclusion that function of Erg25 is critical for nickel tolerance and Erg25 does not simply act as a nickel sink. We have now more extensively discussed these hypotheses in the revised manuscript (see lines 398-415). Briefly, there are a few features that make Erg25 unique relative to other subunits in the complex (Erg26 and Erg27). Erg25 is much more abundant than Erg26 or Erg27, suggesting that Erg25 might have function beyond the one in the complex or that Erg25 is the rate limiting enzyme and so more is critical for Erg25 but not for Erg26/27. The metal binding pocket in Erg25 is unique. None of the histidine rich protein encoding genes in the deletion set we tested affect nickel tolerance and the only reliable nickel-binding protein that we could pull down was Ure7. However, Ure7 is not important for nickel tolerance based on the gene deletion and gene overexpression mutant phenotypes. There are other factors to consider and those would be interesting topics for investigations in the future.

Previously, June Kwon Chung's group found that Erg25 was capable of rescuing the growth of *sre1*Δ and *scp1*Δ exposed to $CoCl_2$. Extra copies of Erg25 were able to reduce 4,4, dimethylsterols in the *scp1*Δ background. They conclude that Erg25 activity is critical for growth on media containing $CoCl_2$. In their work, cobalt caused an increase in Erg25 and Erg3 substrates. This suggests to us, and them, that Ni could be binding to these proteins and inhibiting their function.

We tested overexpression of *ERG3*, which does not confer nickel tolerance. Erg25 acts in complex with Erg26 and Erg27. However, the stoichiometry of these proteins in the cell are not 1:1:1. Erg25 is vastly more abundant in the cell. Yeast two-hybrid screening and *in silico* work predict that Erg25 interacts with proteins involved in vesicular transport, secretory pathway, and cellular localization. The other complex components, Erg26 and Erg27, are not predicted to have such interacting partners [2]. Erg25, therefore, likely has additional functions beyond the complex that may also be influenced by Ni. This allows the *ERG25* overexpression strain, but not the *ERG26* or *ERG27* overexpression strains, to be

resistant to high concentrations of Ni, as well as allowing for the growth of the *sre1*Δ mutant. Testing other ERG genes also indicate their non-consequential impact on nickel tolerance. Thus, Erg25 is uniquely required for nickel tolerance.

The sre1Δ mutant should be reconstituted with the SRE1 gene and key phenotypic analyses performed to confirm that mutant phenotypes are due to loss of SRE1 gene function.

We have overexpressed the N-terminal domain of the *SRE1* gene in the *stp1*Δ background, which partially restored growth on Ni. This spotting assay has been added to Figure 1A in this revised manuscript. The observations that all mutants in that pathway are also defective in growth on nickel support that Sre1 is responsible for the phenotypes observed. The new data showing that nickel activates the cleavage of Sre1 is also consistent with the conclusion that Sre1 pathway is important for nickel tolerance.

The nic1Δ mutant should be directly complemented to ensure that phenotypes are directly related to loss of this gene. In addition, conclusions about nic1 having increased nickel relative to WT are not logical (line 151/152). A putative lower affinity nickel transporter, as proposed by Singh et al. (2013), would not explain increased nickel relative to WT, which presumably has both transporters intact.

The *nic1*Δ strain was confirmed via lack of urease activity (now shown in Supplemental Figure 1B in this revised manuscript). The conclusion about the *nic1*Δ strain having increased Ni relative to WT has been amended to reflect the fact that many divalent cation transporters have been documented to be capable of transporting metals which they do not have high affinity. The Singh et al. (2013) paper has shown that when Ni is added to Christensen Urea Agar, that the *nic1*Δ mutant is capable of urease activity [3]. Since Nic1 is a high affinity Ni transporter, it is likely better capable of regulating Ni diffusion across the membrane. When Nic1 is missing (as in the *nic1*Δ), Ni is able to enter the cell via other metal transporters with likely dis-regulation, resulting in increased cellular Ni in the cell. Ni entry into the cell independent of Nic1 has been observed previously [3]. When *nic1*Δ was plated on media containing urea, the mutant had a growth defect that was rescued when 10µM Ni was added to the media. Likewise, when the *nic1*Δ mutant was plated on Christensen's agar, the mutant shows no urease activity. However, when 10µM Ni was added to the Christensen agar, the mutant was capable of urease activity.

The authors imply that the sre1Δ phenotype can be explained by reduced EBP expression, specifically ERG25, and present data that overexpression of ERG25 increases lipid content. However, this hypothesis should be directly tested by measuring expression of these genes in the sre1Δ mutant (not just by implication of the WT expression changes in nickel and hypoxia) and chemically, for example by adding back the Erg25 intermediate to test for rescue of the phenotype.

In the *sre1*Δ on Ni, when compared to the H99 strain on Ni, the ergosterol biosynthesis genes are some of the most downregulated genes. This shows that Sre1 is required for EBP expression. Comparing to the RNA seq data of the *sre1*Δ mutant versus the WT strain grown in hypoxia conditions revealed major differences between how Ni and hypoxia affect

the transcript abundance of EBP genes. In brief, 12 Erg genes were differentially expressed (defined as │fold change│ ≥2) and 20 total showed altered gene expression (statistically significant) in response to nickel. In the hypoxia condition, however, only 4 Erg genes were differentially expressed, and 7 showed altered gene expression. This data, represented in volcano plots have been added to the manuscript as supplemental figure 3 and the RNA-seq data was added as Tables S4 and S5 in this revised version. The description of this data is summarized now in lines 170-180.

The intermediate 4,4 dimethylzymosterol prior to Erg25 is only commercially available at $917 for 1mg. Like other ergosterol/cholesterol biosynthetic intermediates, the solubility of these compounds is limited (for example the solubility of lanosterol in ethanol is 0.25mg/mL, per Cayman Chemical https://www.caymanchem.com/pdfs/19521.pdf**). In addition, we have permeability concerns as our previous experiments suggest that *Cryptococcus* does not easily take up exogenously added ergosterol.**

Given the hypothesis that nickel and hypoxia exert similar impacts (line 218), comprehensive analysis of the overlap between nickel exposure and hypoxia RNAseq datasets is warranted, beyond the ergosterol pathway.

We believe that nickel and hypoxia exert some similarity and mostly that similarity is limited to the EBP genes (see our response above). We compared the genes shared in the wildtype Ni vs RPMI and hypoxia vs normoxia, and present them as a graph in the new supplemental figure 3 in the revised manuscript. In total, 791 genes are shared between the Ni and hypoxia data set. However, the vast majority of them are not DEGs in both data sets (unique to each condition). There are only 8 DEGs upregulated in both Ni and hypoxia. There are 28 DEGs downregulated in both data sets. EBP genes are highlighted in red. Table S6 includes the expression data as well as a list of shared DEGs.

This result is not unexpected as transcriptome responses to cobalt and hypoxia are also very different [4, 5]. In the two previously referenced studies, June Kwon-Chung's group found limited overlap between genes differentially expressed in low oxygen and cobalt chloride. Nonetheless, they did find that low oxygen and cobalt chloride were both able to impact ergosterol biosynthesis pathway genes as well as iron transport. Previous work in our lab has also identified that hypoxia impacts cellular metabolism, which is independent of Sre1 [6].

The introduction overall is discursive and should be substantially revised to focus on the main topic of study. In particular, paragraph three (on bacterial nickel toxicity, and providing conclusions about the work to be performed etc) should be reconsidered. Contractions should not be used in formal writing. Genus names must be specified the first time they are used. I suggest the authors have a senior colleague review the document throughout for overall logical structure and similar errors in scientific writing.

We thank the reviewer for taking the time to read our manuscript and bringing to our attention about the organization of the introduction. There has not been work done in fungi regarding Ni as a micronutrient. Most work involving fungi and Ni involve bioremediation

studies. Because the species of fungi that these studies use are endophytic fungi and/or non-model fungi, there is lack of molecular studies on nickel response in fungi.

We changed the third paragraph to address the rationale of why we chose *C. neoformans* to pursue these studies. Most of the paragraph involving bacterial species has been removed as the reviewer suggested. We now emphasize that *C. neoformans* is an environmental/soil fungus and thus must have mechanisms in place to tolerate Ni from the natural environment.

What evidence is there that only one enzyme in the cryptococcus genome that binds to nickel? A reference is needed at the very least. A better approach would be to look for evidence of proteins in the Cryptococcus proteome with the capacity to bind nickel in an unbiased way. The physiological relevance of the chosen nickel and other metal concentrations should be justified.

Cryptococcus lacks the homologs of bacterial enzymes that require Ni for activity. The only known nickel-requiring protein is urease. We had utilized two unbiased methods to attempt to identify Ni binding proteins in *Cryptococcus*. In one method, we searched the *C. neoformans* proteome for protein sequences that are enriched in Histidine residues, which are known for their Ni-binding capabilities. We tested several gene deletion mutants corresponding to these proteins of interest and found that none have any phenotype on Ni supplemented RPMI media, nor on media supplemented with Ni-chelator DMG. The phenotypic assay is shown below.

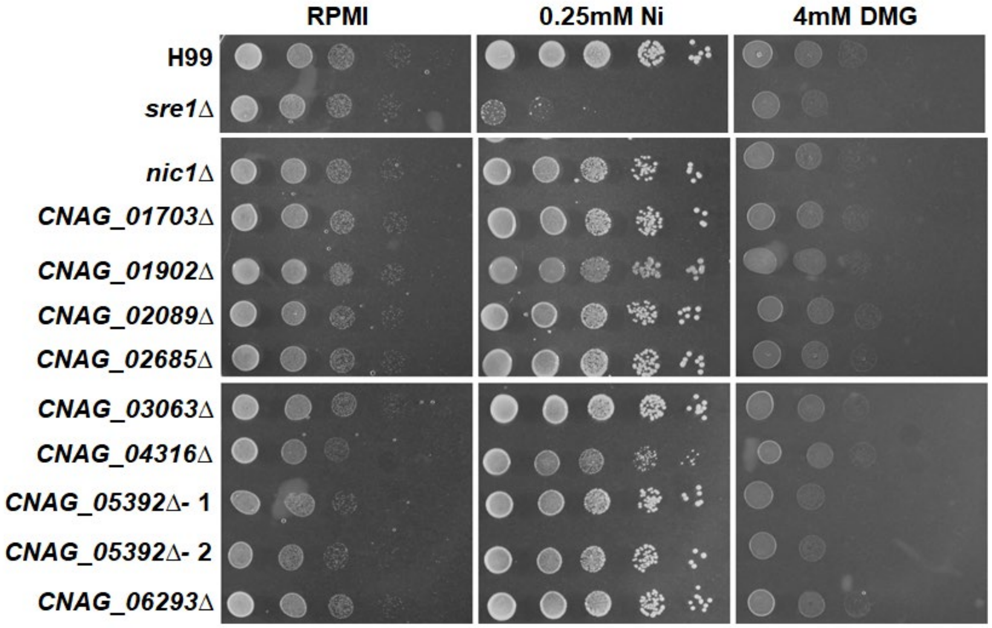

In the second approach, we added cryptococcal protein extract to Ni beads to pull down nickel binding proteins. We were able to reliably pull down Ure7, the known Ni binding chaperone protein that we mentioned earlier. Most of the other proteins that we pulled down in this assay are highly abundant proteins such as Tef1 (transcription elongation factor 1), heat shock proteins, and ribosomal proteins. These proteins are often found in

our negative control samples of Co-IP/MS experiments and are therefore not considered as hits. Based on these observations and previous work by our colleague and coauthor, Dr. Rob Maier, the Ni binding pockets in many proteins are buried deeper within the protein and may not be accessible to the Ni beads. We briefly mentioned these attempts in our discussion in the revised manuscript in case readers may find the information useful. (lines 425-429)

The author summary overstates the link to mammalian data - there is no evidence that mammalian cells use Erg25 to respond to Nickle stress.

We apologize that our original statement (lines 375-377) "This result suggests that Ni also reduces the amount of cholesterol in mammalian cells, similar to its ergosterol reduction effect in fungal cells." caused the reviewer to think that we implied that mammalian cells use Erg25 to respond to Ni stress. We added the following sentence to clarify this. "Whether Erg25 homolog Fet6 is also involved in nickel tolerance in mammals is yet to be tested."

The final two sentences of the discussion (lines 436-439) have also been amended to state "Furthermore, the reduction of sterols in the presence of Ni is conserved in both fungi and mammals. Whether Ni acts on the same targets in fungi and mammals for such an effect is unknown. The current findings could stimulate and guide future investigation to find effective ways to mitigate or prevent nickel toxicity."

Figure 1B The SRE element should be indicated in the Sre1/DNA interaction.

Thanks for the suggestion. This has been added to the figure.

Figure 1C Micronutrients in their salt form should be described in the figure legend.

The salt forms of the metals added to the media have been added to the figure legends where appropriate.

Figure S3 What is Co?

Co refers to cobalt. The figure title has been amended to define this term.

Legends are insufficiently detailed. Unnecessary abbreviations should be avoided in figure legends in general.

We thank the reviewer for noticing this. Details were added to legends where appropriate and abbreviations were removed with the exception of defining labels within figure panels.

The methods section "metal assays" is insufficiently detailed. What is the formulation of RPMI plates (supplier information, formulation, etc)? What concentration of the indicated metals were added and in what formulation? How was appropriate pH confirmed?

We thank the reviewer for the carefulness. For the methods section: supplier and preparation information including how pH adjusted for the RPMI media were added (line 444-446), and the specific metal salts and their concentrations were specified (lines 460-461).

For the legends: details such as which metal salt used were added throughout.

Appropriate statistical methods must be used throughout. Student's t-test is not appropriate for comparing more than 2 groups. It is unclear what comparisons the p-values are reported for. The p-value must be corrected for multiple comparisons.

We agree with the reviewer that t-test is only appropriate for comparison between two groups. We are not comparing more than two groups of data in any of our figures so we find that t-test and Mann Whitney tests are appropriate. To avoid confusion and show that the p-values are only comparing two groups, brackets were added to the figures (particularly figures 1 and 4) in order to distinguish what data is being compared for each set of asterisks.

References cited in this letter

1. Veen M, Stahl U, Lang C. Combined overexpression of genes of the ergosterol biosynthetic pathway leads to accumulation of sterols in *Saccharomyces cerevisiae*. FEMS yeast research. 2003;4(1):87-95.
2. Sukhanova A, Gorin A, Serebriiskii IG, Gabitova L, Zheng H, Restifo D, et al. Targeting C4-demethylating genes in the cholesterol pathway sensitizes cancer cells to EGF receptor inhibitors via increased EGF receptor degradation. Cancer discovery. 2013;3(1):96-111.
3. Singh A, Panting RJ, Varma A, Saijo T, Waldron KJ, Jong A, et al. Factors required for activation of urease as a virulence determinant in *Cryptococcus neoformans*. MBio. 2013;4(3):e00220-13.
4. Chang YC, Bien CM, Lee H, Espenshade PJ, Kwon-Chung KJ. Sre1p, a regulator of oxygen sensing and sterol homeostasis, is required for virulence in *Cryptococcus neoformans*. *Molecular Microbiology*. 2007;64(3):614-29.
5. Lee H, Bien CM, Hughes AL, Espenshade PJ, Kwon-Chung KJ, Chang YC. Cobalt chloride, a hypoxia-mimicking agent, targets sterol synthesis in the pathogenic fungus *Cryptococcus neoformans*. *Molecular microbiology*. 2007;65(4):1018-33.
6. Zhao Y, Lin X. A PAS Protein Directs Metabolic Reprogramming during Cryptococcal Adaptation to Hypoxia. Mbio. 2021;12(2):e03602-20.
